# Endoscopic excision of a unilateral sinonasal hamartoma

**DOI:** 10.1093/jscr/rjag287

**Published:** 2026-04-22

**Authors:** Colten Witte, David J Dillard, Shaynie Segal, James K Fortson

**Affiliations:** Otolaryngology, Sleep and Sinus Centers of Georgia, 1990 Riverside Pkwy, Lawrenceville, GA 30043, United States; Otolaryngology, Sleep and Sinus Centers of Georgia, 1990 Riverside Pkwy, Lawrenceville, GA 30043, United States; Otolaryngology, Sleep and Sinus Centers of Georgia, 1990 Riverside Pkwy, Lawrenceville, GA 30043, United States; Otolaryngology, ENT Associates of South Atlanta, 1151 Cleveland Ave STE C, East Point, GA 30344, United States

**Keywords:** sinonasal hamartoma, unilateral nasal mass, endoscopic excision, respiratory epithelial adenomatoid hamartoma, chronic rhinosinusitis

## Abstract

Sinonasal hamartomas are rare, benign developmental lesions of the nasal cavity that may mimic inflammatory polyps or neoplastic processes, particularly when presenting as unilateral nasal masses. Accurate diagnosis is essential to prevent unnecessary aggressive management. We report the case of a 43-year-old male with a history of chronic rhinosinusitis who presented with progressive right-sided nasal obstruction and was found to have a unilateral nasal cavity mass. The patient underwent endoscopic excision of the lesion with clear margins. Gross pathologic examination revealed irregular tan-pink mucosal and cartilaginous tissue fragments measuring up to ~2.5 cm. Histopathologic analysis demonstrated disorganized sinonasal elements without cytologic atypia, invasive growth, or malignant features, consistent with a sinonasal hamartoma. The postoperative course was uncomplicated, and no further intervention was required. This case highlights the importance of considering hamartomas in the differential diagnosis of unilateral nasal masses and demonstrates that complete endoscopic excision is both diagnostic and curative.

## Introduction

Hamartomas are benign, non-neoplastic lesions composed of disorganized but mature tissue elements native to the site of origin. Within the sinonasal tract, hamartomas are rare and may arise from epithelial, mesenchymal, or mixed tissue components. Their clinical presentation frequently overlaps with that of inflammatory polyps, inverted papillomas, and malignant sinonasal tumors, particularly when unilateral in nature [1].

Unilateral nasal masses warrant careful evaluation due to the increased likelihood of neoplastic pathology. While imaging and endoscopic appearance may suggest benign disease, definitive diagnosis often relies on histopathologic examination. Awareness of sinonasal hamartomas is important to avoid overtreatment, including unnecessary oncologic resection or adjuvant therapy [2]. We present a case of a sinonasal hamartoma identified following endoscopic excision of a unilateral nasal cavity mass and discuss its diagnostic and management considerations.

## Case report

A 43-year-old male with a history of chronic sinusitis presented with progressive right-sided nasal obstruction. He denied epistaxis, facial pain, anosmia, or visual symptoms. Computed tomography of the sinuses was performed and revealed nasal septal deviation to the left and olfactory cleft widening of the right nasal cavity. A mass ~3.5 × 1.0 × 1.0 cm was appreciated in the right inferior middle meatus with moderate bilateral maxillary and ethmoid rhinosinusitis.

Given his unilateral presentation and persistent symptoms, surgical excision was recommended for both diagnostic and therapeutic purposes. Using nasal endoscopy and image guidance, the right middle turbinate was medialized. The lateral nasal wall was injected with lidocaine with 1:100,000 epinephrine. A fleshy mass was found to be pedunculated along the medial maxillary wall, extending from just below the natural os to the posterior border of the fontanelle. A sickle knife and endoscopic scissors were used to separate and remove the mass in two fragments. The cut edge was cauterized, and the remainder of the endoscopic sinus procedure was completed without complications.

**Figure 1:**
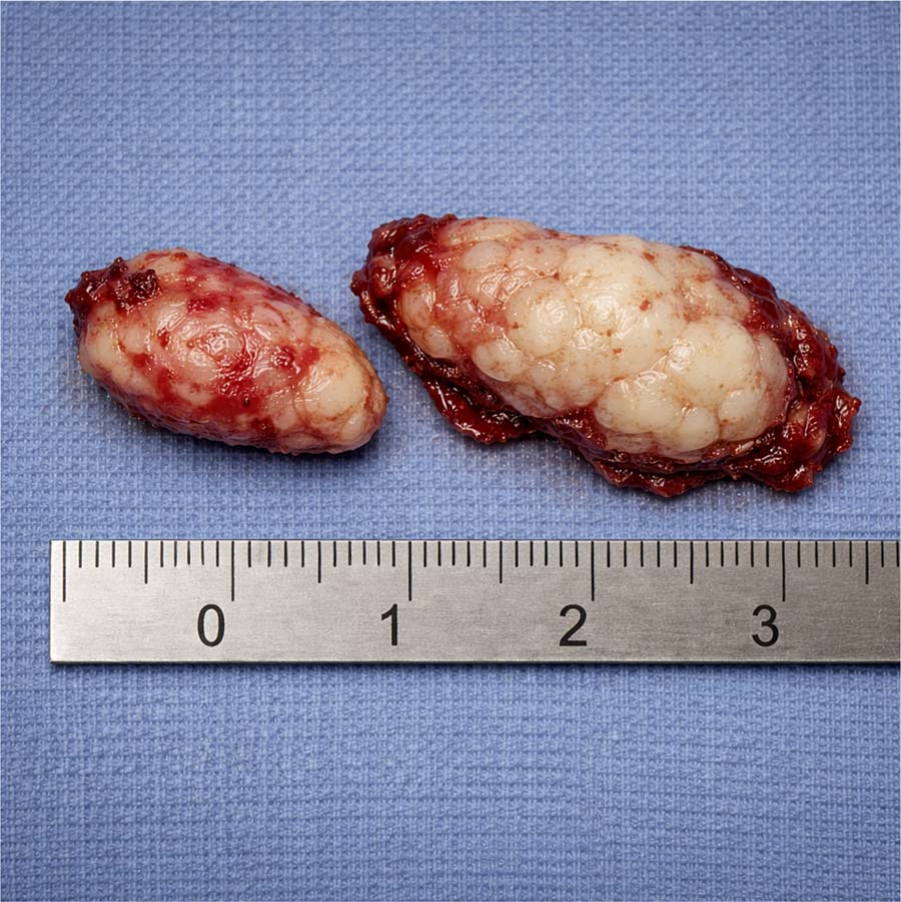
Gross specimen demonstrating irregular tan-pink fragments of mucosal and cartilaginous tissue measuring ~2.0 and ~ 2.5 cm.

Gross pathologic examination revealed two irregular tan-pink fragments of mucosal and cartilaginous tissue measuring ~2.0 cm and ~ 2.5 cm in greatest dimension (Fig. 1). Microscopic histologic evaluation demonstrated disorganized but benign sinonasal tissue elements, including submucosal gland-like proliferation lined with benign ciliated respiratory epithelium, thickened basement membranes, and underlying stromal components. There was no evidence of cytologic atypia, increased mitotic activity, necrosis, invasive growth, or malignant transformation. These findings were consistent with a sinonasal hamartoma.

The patient’s postoperative course was uncomplicated. Nasal obstruction resolved, and no additional surgical or medical therapy was required. At follow-up 6 weeks later, there was no evidence of recurrence, and the cauterized base was healing as expected. The patient was instructed to return to the office if any complications arose.

## Discussion

Sinonasal hamartomas present a diagnostic challenge because they can closely resemble conditions with different prognoses and management, especially in unilateral cases where concern for inverted papilloma or malignancy is warranted [2, 3]. Unilateral obstruction with a polypoid mass is nonspecific, as inflammatory polyps, respiratory epithelial adenomatoid hamartomas, inverted papillomas, and malignancies can share similar symptoms and endoscopic findings [2, 3]. Therefore, clinicians should prioritize assessment of laboratory findings and management implications over appearance alone.

A contemporary approach is to view sinonasal hamartomas as a spectrum of related lesions. Respiratory epithelial adenomatoid hamartoma, the most common subtype, features benign glandular proliferation lined by ciliated respiratory epithelium, absence of dysplasia, and noninvasive growth [1, 4, 5]. Chondro-osseous respiratory epithelial adenomatoid hamartoma is a less common variant with chondro-osseous stromal elements, but case series and reviews indicate similarly benign behavior and excellent outcomes after complete excision [6, 7]. Considering these lesions in the differential diagnosis encourages surgeons and pathologists to include hamartoma for unilateral disease.

Imaging can refine preoperative suspicion but cannot replace histology. One of the most reproducible radiographic associations for respiratory epithelial adenomatoid hamartoma is olfactory cleft widening with soft tissue opacification [8–10]. Prior work highlights an olfactory cleft width threshold that increases suspicion and can prompt intentional sampling or complete excision of tissue in the olfactory cleft region, particularly in patients otherwise presumed to have chronic inflammatory disease [8–10]. A systematic review further supports the association of olfactory cleft widening and reports that many cases are identified in patients undergoing surgery for chronic rhinosinusitis or nasal polyposis [11]. However, because imaging overlap persists across inflammatory and neoplastic conditions, and because unilateral disease remains a clinical red flag, imaging should be treated as probabilistic support rather than a definitive discriminator [2, 3, 9].

The link between chronic inflammation and hamartoma formation is receiving increased attention. Reviews frequently report respiratory epithelial adenomatoid hamartoma coexisting with chronic rhinosinusitis and nasal polyposis, suggesting that chronic mucosal inflammation may contribute to lesion development or recognition in surgical cohorts [4, 5]. Recent observational studies have examined inflammatory endotypes in surgically treated cases and support the idea that inflammatory biology may affect lesion distribution and bilaterality [10]. Although causality is unproven, it reminds clinicians that a unilateral polypoid lesion in chronic sinusitis may be benign and supports counseling that symptom-driven excision is usually definitive once benign pathology is confirmed [4, 5, 10, 11].

Distinguishing hamartomas from inverted papilloma histopathologically is essential, as it determines surgical approach and follow-up. Inverted papilloma has a significant risk of recurrence and potential for malignant transformation, requiring careful management of the attachment site and extended surveillance [12]. In contrast, hamartomas are benign and typically cured by complete endoscopic excision, with rare recurrence when fully resected [1, 11]. Accurate diagnosis helps avoid both overtreatment, such as unnecessary oncologic surgery or adjuvant therapy, and undertreatment of true papilloma or malignancy [1, 12]. Although this case did not warrant complex endoscopic techniques, masses in other sinuses, such as frontal, require techniques such as the Draf III procedure [13].

Structured evaluation algorithms for unilateral sinonasal disease support balanced decision making [2, 3]. Foundational studies show that combining imaging, endonasal endoscopy, and biopsy improves preoperative diagnostic accuracy and enables minimally invasive management in cases without bone destruction [3]. Recent reviews also highlight the importance of standardized assessment due to the broad differential [2]. The present case reflects these principles: complete endoscopic excision provides diagnostic histological findings and curative symptom resolution, avoiding unnecessary escalation after benign pathology is confirmed.
